# Relation of antioxidant status at admission and disease severity and outcome in dogs naturally infected with *Babesia canis canis*

**DOI:** 10.1186/s12917-017-1020-9

**Published:** 2017-04-24

**Authors:** Martina Crnogaj, José Joaquin Cerón, Iva Šmit, Ivana Kiš, Jelena Gotić, Mirna Brkljačić, Vesna Matijatko, Camila Peres Rubio, Nada Kučer, Vladimir Mrljak

**Affiliations:** 1 Clinic for Internal Diseases, Faculty of Veterinary Medicine, University of Zagreb, Zagreb, Croatia; 2 Department of Animal Medicine and Surgery, Faculty of Veterinary Medicine, University of Murcia, 30100 Espinardo, Murcia, Spain

**Keywords:** Babesiosis, Dog, Antioxidant status, Glutathione peroxidase (GPx), Superoxide dismutase (SOD), Catalase, Total antioxidant status (TAS)

## Abstract

**Background:**

Canine babesiosis is caused by species of the *Babesia* genus and has become an emerging disease worldwide. To the authors’ knowledge there are no reports in which antioxidants have been analyzed in different presentations of canine babesiosis or in which the prognostic value of antioxidants has been studied. The aim of this study was to evaluate whether oxidative stress could be related to the severity and outcome of canine babesiosis. For this purpose a profile consisting of four antioxidant biomarkers (superoxide dismutase - SOD, glutathione peroxidase - GPx, catalase, total antioxidant status - TAS) and malondialdehyde - MDA as an oxidant biomarker (previously evaluated, here studied for comparative purposes) were evaluated in dogs with canine babesiosis of different clinical severity and outcomes.

**Results:**

The study was conducted with a sample of 40 dogs suffering from babesiosis (further divided into uncomplicated, one complication and multiple organ dysfunction syndrome - MODS group) and 30 healthy dogs (control group). Additionally, the babesiosis group was divided according to the anaemia into non-anaemic, mildly anaemic, moderately anaemic and severely anaemic dogs. The results of our study showed significantly decreased SOD, catalase and TAS values in diseased dogs compared to controls, while there were no significant differences in GPx between these groups. Dogs that developed MODS showed lower activities of SOD and GPx and higher MDA values compared to dogs with uncomplicated babesiosis as well as with dogs that developed one complication. Superoxide dismutase, catalase and GPx were negatively correlated whereas MDA was positively correlated with the lethal outcome of the disease. Furthermore, this study detected more pronounced decrease in antioxidant biomarkers (SOD, GPx and catalase) in dogs with moderate anaemia compared to those with mild anaemia.

**Conclusions:**

The results of this study showed changes in biomarkers related to the antioxidant status of dogs naturally infected with *B. canis canis*. These biomarkers could be used as indicators of disease severity and outcome in dogs suffering from babesiosis.

**Electronic supplementary material:**

The online version of this article (doi:10.1186/s12917-017-1020-9) contains supplementary material, which is available to authorized users.

## Background

Canine babesiosis is a tick born multisystemic disease with worldwide significance [1]. *Babesia spp*. are well-known intraerythrocytic parasites that cause disease in domestic and wild animals. Babesiosis in dogs is caused by species of the *Babesia* genus, which are divided into large babesia including *Babesia canis* and *Babesia sp. (Coco)* and small babesia including *Babesia gibsoni (B. gibsoni), Babesia conradae (B. conradae)* and *Babesia microti-*like piroplasms, also called *Babesia vulpes sp. nov.* There are three genetically distinct subspecies of *Babesia canis*: *Babesia canis canis (B.canis canis), Babesia canis vogeli (B.canis vogeli)* and *Babesia canis rossi (B. canis rossi)* [2–6].

Host response to infection and other forms of tissue injury in humans, as well as in dogs, have been termed the systemic inflammatory response syndrome (SIRS). This inflammatory response can frequently be accompanied by oxidative injury to one or more organ systems in the body leading to multiple organ dysfunction syndrome (MODS), which occurs in canine babesiosis and is related to poor prognosis [2, 7, 8]. Considering the fact that sepsis is defined as SIRS due to a confirmed infection (bacterial, viral, fungal or protozoal) canine babesiosis can be classified as protozoal sepsis [8–13].

Based on clinical manifestations, babesiosis can be classified as uncomplicated (without any organ dysfunction) and complicated form (involving one or various organ dysfunctions such as kidney, liver, lung, etc.) [14, 15]. Although various mechanisms have been suggested to cause both forms of babesiosis, recent studies have indicated that much of the disease process could be explained by host inflammatory responses to the parasite, rather than the parasite itself [16, 17]. The hypothesis that cytokines may also have an influence on the severity of babesiosis in dogs was investigated in several studies with the implication that a mixed cytokine response is present in dogs with babesiosis, and that an excessive pro-inflammatory response may result in a poor outcome [18]. An imbalance in host regulation of the pro-inflammatory systemic response and a compensatory modulating response can frequently be accompanied by oxidative injury in one or more organ systems in the body leading to progression from SIRS to MODS in septic patients with a fatal outcome in certain cases [18–21].

Regardless of its pathogenesis, any tissue damage, if severe enough, induces the release of proinflammatory mediators, including reactive oxygen species (ROS) and reactive nitrogen species (RNS), which are powerful oxidants and nitrating species that can inactivate enzymes and initiate lipid peroxidation and nitration, which in turn leads to free-radical chain reactions that further damage proteins, membranes and nucleic acids [22, 23].

An antioxidant is defined as “any substance that, when presented at low concentration compared to those of an oxidizable substrate (proteins, lipids, carbohydrates and DNA), significantly delays, or prevents oxidation of that substrate”, thus protecting the body by elimination of the superoxide anion and hydroperoxides that may oxidize cellular substrates, and preventing a chain reaction of the destructive effects of free radicals [21, 24–26]. Superoxide dismutase (SOD), catalase and glutathione peroxidase (GPx) are the primary intracellular antioxidants and as such act as protective mechanisms during elevated oxidative stress. Total antioxidant status (TAS) evaluates the antioxidant activity of the organism in a global way [27, 28].

Oxidative stress is caused by an imbalance between oxidants and antioxidants and may occur at the level of cells, tissues, or even the whole organism [29]. In recent years, various studies have evaluated the role of oxidative stress and lipid peroxidation in the pathogenesis of babesiosis in different animal species [30–37]. However, to the authors’ knowledge there are no studies in which antioxidant biomarkers have been analyzed in different presentations of canine babesiosis or their prognostic value studied. Furthermore, there are no studies in which the antioxidants have been analyzed in canine babesiosis caused by *B. canis canis.*

One of the main features of babesiosis is destruction of red blood cells resulting in hemolytic anaemia. However, the pathogenesis of anaemia in babesiosis has not yet been fully elucidated [38]. The quantity of the destroyed erythrocytes is usually much higher than the degree of parasitaemia, suggesting that non-parasited erythrocytes may also be damaged [39]. Some of the proposed mechanisms responsible for this phenomena could be: sequestration of infected erythrocytes in microcirculation, decreased erythrocyte deformability, hemodilution and destruction of red blood cells due to the effects of oxidative stress [17, 40–43].

The hypothesis of the current study is that the antioxidant response in dogs infected with *B. canis canis* could change depending on the severity of the clinical presentation, and that these changes could be related to the outcome of the disease. For this purpose a profile consisting of four antioxidant biomarkers, GPx, SOD, catalase and TAS, were measured in dogs with canine babesiosis, and changes in these markers were compared between healthy dogs and dogs with babesiosis, between dogs with and without complications, as well as between survivors and non-survivors.

## Methods

### Animals

This study was performed with 70 dogs that were divided into two groups: babesiosis and control group. All dogs were admitted to the Clinic for Internal Diseases, Faculty of Veterinary Medicine, University of Zagreb, Croatia and owner consent was obtained for all dogs included in this study as part of a routine clinical protocol.

All dogs included in the study were clinically examined and blood samples were collected from the cephalic vein on the day of admission for haematology and biochemical analysis: blood urea nitrogen (BUN), creatinine, total protein (TP), albumin, alanine aminotransferase (ALT), alkaline phosphatase (AP), aspartate aminotransferase (AST), γ-glutamyl transferase (GGT), glucose, total bilirubin and creatine phosphokinase (CPK).

The babesiosis group consisted of 40 dogs naturally infected by *B. canis canis* showing clinical signs of acute babesiosis. Dogs in this group were aged between 1 and 13 years, of various breeds and both genders (27 male dogs: 67.5%, 13 female dogs: 32.5%). The diagnosis was confirmed by demonstration of the parasite within infected erythrocytes in blood smears stained with May-Grünwald Giemsa solution and polymerase chain reaction (PCR) analysis performed as previously described [44].

On the basis of clinical manifestations and laboratory data the affected dogs were divided into two groups: uncomplicated and complicated babesiosis, whereas the complicated group was further subdivided into those with one complication and the MODS group, due to the criteria listed in Table 1 [8, 11]. Additionally, the babesiosis group was divided according to the degree of anaemia (on the basis of haematocrit - HCT percentage) into non-anaemic: HCT >37%, mildly anaemic: 30–37%, moderately anaemic: 18–29% and severely anaemic: <18% dogs [45]. All the dogs from the babesiosis group developed SIRS according to a previous described criteria [46].

**Table 1:** Criteria used for distribution of affected dogs into subgroups

Uncomplicated babesiosis *N* = 29	Complicated babesiosis dogs that developed at least one of listed complication *N* = 11		
Dogs without any listed complication	Complication		Criteria
	Renal dysfunction		Creatinine >180 μmol/L
	Respiratory system dysfunction		Dyspnoea with typical nasal discharge or radiographic evidence of pulmonary oedema
	Hepatic dysfunction		ALT >176 U/L, AP >360 U/L, and Bilirubin* >100 μmol/L
	Muscular involvement		CPK > 600 U/L
	Central nervous system dysfunction		a modified Glasgow coma scale <9**
	Additional complication***	Secondary infection	WBC > 17 × 10^9/^L (neutropenia with lymphopenia)
		Pancreatitis	Positive SNAP cPL test

*cPL* canine pancreas-specific lipase

*We included a total bilirubin serum concentration greater than 100 μmol/L as an additional criterion for hepatic dysfunction [62]

**Modified Glasgow coma scale: Welzl et al. [12]

***Pancreatitis [13] as well as signs of secondary infection were not considered as main complication, they were only mentioned (since being detected) as an additional complication

The control group consisted of 30 clinically healthy dogs of various breeds having a similar age (between 1 and 13 years) and gender distribution (19 male dogs: 63.3%, 11 female dogs: 36.7%) analogous to the infected dogs. The dogs were deemed healthy on the basis of history, clinical examination and laboratory data.

All dogs with confirmed babesiosis received a single dose (6.6 mg/kg of body weight) of imidocarb dipropionate (Imizol® 12%, Schering-Plough), subcutaneously. Dogs with complications received a standard additional therapy, according to clinical condition and type of complication. The therapy included: intravenous fluids (crystalloids ± colloids), number of dogs (*N*) = 11, oxygen supplementation via intranasal tubes or oxygen cage, *N* = 3, analgesia (fentanyl transdermal patch, according to patient body weight), *N* = 5, and intravenous antibiotics (amoxicillin clavulanic acid: 22 mg/kg intravenously every 8 h as monotherapy, or in combination with enrofloxacin 10 mg/kg intravenously every 12 h), *N* = 7.

### Blood analyses

Samples were placed in tubes with ethylenediaminetetraacetic acid (EDTA) for haematological analysis, tubes with lithium heparin for analyzing SOD, GPx and catalase and tubes with no anticoagulant (centrifuged at 1500 × g at 4 °C for 10 min) for analyzing biochemistry profile, TAS and MDA. After sampling, blood was promptly analyzed for haematological analysis and routine biochemistry panel. Since acute pancreatitis has previously been identified as a potential complication of canine babesiosis [47] we also performed a SNAP cPL (canine pancreas-specific lipase) test (Idexx Laboratories, Westbrook) in order to confirm the presence of pancreatitis as a concurrent complication in 14 dogs that showed elevated lipase activity in our routine biochemistry profile.

After the initial analysis samples were stored at −80 °C until antioxidant measurements. The activities of GPx, SOD and catalase were determined in whole blood. Concentrations of TAS and MDA were determined from serum samples.

Activity of GPx was measured with a commercially available kit (Ransel test kit, Randox Laboratories Ltd. G.B.) based on the method of Paglia and Valentine [48]. SOD activity was measured with a commercial kit (Ransod test kit, Randox Laboratories Ltd. G.B.). Catalase activity was determined according to Johansson and Borg [49]. The activities of GPx and catalase were expressed as liter of whole blood (U/L). The activity of SOD was expressed as milliliter of whole blood (U/mL). Determination of TAS was through use of commercial test reagents (Randox test kit, Randox Laboratories Ltd. GB) according to the manufacturer’s instructions. The concentration of TAS was expressed in mmol/L.

The concentration of MDA was measured with the method of Trotta et al. [50]. Absorbance was measured at 523 nm on a Thermospectronic Helios delta spectrophotometer (Unicam, Cambridge, UK). The concentration of MDA was expressed in μmol/L.

### Statistical analysis

Pearson X^2^ test was used to assess the significance of gender distribution between control and babesiosis groups. In order to test the difference between ages of control and babesiosis groups we conducted t-test for independent samples. In order to assess the normality of data distribution, Kolgomorov-Smirnov test was used. Given the non-parametric distribution of quantitative values, descriptive statistics were performed and the results are presented as median and interquartile range. Differences in analytes between groups were analyzed by Mann-Whitney U-test, with a *P* value <0.05 considered significant. The correlation between different biomarkers as well as between biomarkers and other laboratory parameters were assessed by Sperman rank correlation test. The relationship between oxidative status markers and outcomes was assessed using Tau-b correlation. The computer software IBM SPSS Statistics version 19.0.0.1. was used for analysis (www.spss.com).

## Results

Pearson X^2^ value = 0.132 with a *P* value of 0.716 indicated there were no significant differences in gender between the babesiosis and the control group. T-value = 0.29 with *P* value >0.05 indicated there was no significant difference in age between the tested groups.

Depression (37/40), fever (35/40) and anorexia (32/40) were the most prevalent clinical signs at admission. Forty cases fulfilled the selection criteria for acute canine babesiosis and were included in the study. The presence of *B. canis canis* species was confirmed by PCR analysis for all 40 dogs. Uncomplicated babesiosis was diagnosed in 29 (72.5%) and complicated babesiosis in the remaining 11 dogs (27.5%). In dogs with complicated babesiosis, 6 (54.55%) had single organ dysfunction and 5 (45.45%) dogs had MODS. The observed complications were renal dysfunction (5/11), hepatic dysfunction (5/11), muscular involvement (7/11), respiratory system dysfunction (2/11) and central nervous system (CNS) dysfunction (1/11). In dogs that developed MODS, one had developed 4 organ dysfunction (namely: kidney, liver, CNS and muscles), two had 3 organ dysfunction (kidney, muscle and liver; kidney, muscle, respiratory system) while two had 2 organ dysfunction (kidney and muscle; liver and respiratory system). Five out of 14 dogs tested with the SNAP cPL test had positive results, four of which were classified in the MODS group, whereas one was classified in the group with one complication (namely, muscular involvement). Two out of 40 dogs were presented with haematology findings (elevated white blood cell number with marked lymphopenia and neutrophilia) on the basis of which they were considered to have secondary infection. Treatment was successful in all dogs without development of any complications as well as in 7 cases of complicated babesiosis, while 4 dogs with MODS died despite treatment (mortality rate 10%). Anaemia was detected in 23 dogs. Anaemia was severe only in 1/40 (2.5%), moderate in 6/40 (15%) and mild in 16/40 (40%) dogs.

Thirty dogs fulfilled the selection criteria for healthy animals and were included in the study as a control group.

Descriptive statistics of biochemistry parameters (urea, creatinine, bilirubine, ALT, AP, CPK), and haematology parameters (red blood cells - RBC, HCT, white blood cells - WBC and platelets - PLT) are shown in Additional file 1. Descriptive statistics of oxidative biomarkers (SOD, catalase, GPx, TAS and MDA) are shown in Tables 2, 3, 4 and 5.

**Table 2:** Values of biomarkers of oxidative stress in control and babesiosis group

	Control (*N* = 30)	Babesiosis (*N* = 40)
SOD (U/mL)	**0.26** ^**a**^ (0.23–0,30)	**0.18** ^**b**^ (0.15–0.22)
GPx (U/L)	**63,761.6** ^**a**^ (54,664.82–74,960.58)	**60,366.21** ^**a**^ (44,386.68–73,140.63)
Catalase (U/L)	**7992.15** ^**a**^ (5944.98–11,082.35)	**4902.49** ^**b**^ (2000.96–6790.53)
TAS (mmol/L)	**1.25** ^**a**^ (1.17–1.32)	**0.97** ^**b**^ (0.9–1.13)
MDA (μmol/L)	**2.31** ^**b**^ (1.90–2.80)	**4.5** ^**a**^ (3.11–5.67)

Median (25–75 percentile). Different superscripted small letters (a-b) in each row indicate significant differences (*P* < 0.001) and the same superscripted letters indicate no statistical difference. Also, a → b signifies highest to lowest value

**Table 3:** Values of biomarkers of oxidative stress in control and babesiosis subgroups

	Control (*N* = 30)	Uncomplicated (*N* = 29)	Complicated (*N* = 11)	
			One complication (*N* = 6)	MODS (*N* = 5)
SOD (U/mL)	**0.26** ^**a**^ (0.23–0,30)	**0.18** ^**b**^ (0.17–0.23)	**0.17** ^**b**^ (0.15–0.24)	**0.07** ^**c**^ (0.04–0.10)
GPx (U/L)	**63,761.6** ^**a**^ (54,664.82–74,960.58)	**63,761.79** ^**a**^ (57,987.78–76,193.72)	**58,446.62** ^**a**^ (38,885.96–74,709.27)	**29,958.11** ^**b**^ (11,456.91–36,238.04)
Catalase (U/L)	**7992.15** ^**a**^ (5944.98–11,082.35)	**5702.03** ^**b**^ (3338.82–6697.92)	**5319.77** ^**ab**^ (1398.63–9084.15)	**1265.76** ^*c^ (1081.47–2145.59)
TAS (mmol/L)	**1.25** ^**a**^ (1.17–1.32)	**0.97** ^**b**^ (0.89–1.08)	**1.02** ^**b**^ (0.89–1.13)	**1.15** ^**b**^ (0.85–1.7)
MDA (μmol/L)	**2.31** ^**c**^ (1.90–2.80)	**4.50** ^**b**^ (2.99–5.24)	**4.44** ^**b**^ (2.99–5.54)	**10.5** ^**a**^ (7.03–13.66)

Median (25–75 percentile). Different superscripted small letters (a-c) in each row indicate significant differences (*P* < 0.05) and the same superscripted letters indicate no statistical difference. Also, a → c signifies highest to lowest value

*There was no significant difference between MODS and one complication group in catalase activity

**Table 4:** Values of biomarkers of oxidative stress in non-anaemic and anaemic babesiosis group

	Non-anaemic (N 17)	Anaemic (N 23)
SOD (U/mL)	**0.22** ^**a**^ (0.19–0.25)	**0.16** ^**b**^ (0.14–0.17)
GPx (U/L)	**63,761.79** ^**a**^ (59,413.29–76,193.72)	**55,268.09** ^**a**^ (36,798.41–71,138.54)
Catalase (U/L)	**4983.58** ^**a**^ (3072.30–7022.41)	**4821.39** ^**a**^ (1717.07–6350.30)
TAS (mmol/L)	**0.97** ^**a**^ (0.91–1.13)	**0.98** ^**a**^ (0.86–1.13)
MDA (μmol/L)	**3.21** ^**b**^ (2.41–4.50)	**5.18** ^**a**^ (4.44–6.97)

Median (25–75 percentile). Different superscripted small letters (a-b) in each row indicate significant differences (*P* < 0.05) and the same superscripted letters indicate no statistical difference. Also, a → b signifies highest to lowest value

**Table 5:** Values of biomarkers of oxidative stress in dogs with babesiosis according to severity of anaemia

*	Non-anaemic (N 17)	Mildly anaemic (N 16)	Moderately anaemic (N 6)
SOD (U/mL)	**0.22** ^**a**^ (0.19–0.25)	**0.17** ^**b**^ (0.15–0.18)	**0.10** ^**c**^ (0.07–0.14)
GPx (U/L)	**63,761.79** ^**a**^ (59,413.29–76,193.72)	**64,598.96** ^**a**^ (49,248.25–77,186.53)	**36,238.04** ^**b**^ (23,134.80–42,991.35)
Catalase (U/L)	**4983.58** ^**a**^ (3072.30–7022.41)	**5842.74** ^**a**^ (2423.70–7178.58)	**2145.59** ^**b**^ (1199.79–5087.84)
TAS (mmol/L)	**0.97** ^**a**^ (0.91–1.13)	**0.97** ^**a**^ (0.87–1.08)	**1.05** ^**a**^ (0.75–1.35)
MDA (μmol/L)	**3.21** ^**b**^ (2.41–4.50)	**5.09** ^**a**^ (4.41–6.23)	**7.9** ^**a**^ (4.39–12.63)

Median (25–75 percentile). Different superscripted small letters (a-c) in each row indicate significant differences (*P* < 0.05) and the same superscripted letters indicate no statistical difference. Also, a → c signifies highest to lowest value

*Since there was only one severely anaemic dog statistic analysis could not be preformed

The activities of SOD and catalase as well as TAS concentration were significantly lower (*P* < 0.001) in diseased dogs compared with the control group. There was no significant difference (*P* > 0.05) in GPx activity between diseased dogs and controls. The concentration of MDA was significantly increased (*P* < 0.001) in diseased dogs in comparison to the control group (Table 2).

No significant changes were found in investigated biomarkers between dogs with one complication and dogs with uncomplicated babesiosis. However, dogs with MODS showed significantly lower (*P* < 0.01) activities of catalase, SOD and GPx and significantly higher (*P* < 0.01) MDA concentrations compared to dogs with uncomplicated babesiosis. Moreover, dogs with MODS showed significantly lower activities of SOD (*P* < 0.01) and GPx (*P* < 0.05) and significantly higher (*P* < 0.05) MDA concentrations compared to dogs that developed only one complication, however there was no statistical significance detected in catalase activity between these groups (Table 3). Concentrations of TAS were higher in dogs with MODS compared to dogs with uncomplicated babesiosis as well as with dogs that developed only one complication, but detected differences were not statistically significant. Correlations between biochemistry parameters and investigated biomarkers are shown in Table 6. There was a significant negative correlation between antioxidants (SOD, GPx and catalase) and bilirubin but a significant positive correlation between MDA and bilirubin. Furthermore, there was a strong negative correlation of SOD and GPx with CPK and a positive correlation between MDA and urea. In addition, there was a significant negative correlation between GPx and AP.

**Table 6:** Correlations between oxidative biomarkers and the other analytes measured in dogs with babesiosis

Sperman rank correlation coefficient	TAS (mmol/L)	MDA (μmol/L)	SOD (U/mL)	GPx (U/L)	Catalase (U/L)
HCT (%)	-0.62	−0.559**	0.740**	0.531**	0.190
Urea (mmol/L)	−0.073	0.500**	−.0.280	−0.292	−0.332*
Creatinine (μmol/L)	−0.211	0.209	−0.160	0.086	−0.195
Bilirubine (μmol/L)	−0.107	0.342*	−0.330*	−0.476**	-0.458**
ALT (U/L)	-0.237	0.026	0.001	0.065	-0.103
AP (U/L)	0.123	−0.084	−0.270	−0.386*	-0.153
CPK (U/L)	-0.087	0.266	−0.410**	−0.438**	-0.219

*P < 0.05

**P < 0.01

The lethal outcome of the disease was significantly negatively correlated with SOD, GPx, and catalase, and significantly positively correlated with MDA and TAS (Table 7).

**Table 7:** Correlations of biomarkers of oxidative stress with the outcome in dogs with babesiosis

Nominal correlation coefficient		TAS (mmol/L)	MDA (μmol/L)	SOD (U/mL)	GPx (U/L)	Catalase (U/L)
Lethal outcome	Tau-b	0.264	0.222	−0.316	−0.301	-0.227
	P	**0.005**	**0.019**	**0.001**	**0.001**	**0.016**

Correlations between the investigated biomarkers are shown in Table 8. There was a significant negative correlation between MDA and antioxidants (SOD, GPx, catalase). A significant positive correlation was detected between GPx and SOD as well as between GPx and catalase.

**Table 8:** Correlations between biomarkers of oxidative stress in dogs with babesiosis

Sperman rank correlation coefficient	TAS (mmol/L)	MDA (μmol/L)	SOD (U/mL)	GPx (U/L)	Catalase (U/L)
TAS (mmol/L)	X	−0.021	−0.135	−0.149	0.118
MDA (μmol/L)		X	**−.435** ^**^	**−.328** ^*^	**−.321** ^*^
SOD (U/mL)			X	**.526** ^**^	0.285
GPx (U/L)				X	**.380** ^*^
Catalase (U/L)					X

*P < 0.05

**P < 0.01

Values of SOD were significantly lower (*P* < 0.001) whereas values of MDA were significantly higher (*P* < 0.001) in anaemic dogs with babesiosis compared to non-anaemic dogs (Table 4). Furthermore, considering the severity of anaemia, the activities of SOD, GPx and catalase were significantly lower (*P* < 0.01) in moderately anaemic dogs compared to mildly anaemic. There was no significant difference in TAS concentration between anaemic and non-anaemic dogs (Table 4) as well as between different degrees of anaemia (Table 5). Also, there was a significant positive correlation of HCT with GPx and SOD, whereas significant negative correlation of HCT with MDA (Table 6).

## Discussion

The aim of this study was to evaluate the possible changes of four antioxidant biomarkers (SOD, GPx, catalase and TAS) depending on the severity of canine babesiosis at admission and their correlation with the outcome. In addition, the relationship of these markers with anaemia was evaluated. An oxidant biomarker (MDA) previously evaluated in canine babesiosis was studied for comparative purposes [32].

Considering noted marked differences in oxidative stress between males and females in previous studies [51, 52] the gender representation in the control group was purposeful, nonrandom sample. Accordingly, in the current study there was a proportional gender representation between the babesiosis and the control group.

In the present study, in general, a decrease in the antioxidant biomarkers in dogs with babesiosis was observed. These results agree with those reported earlier in sheep suffering from babesiosis [34, 53], in cattle suffering from theileria [31, 54] and in people suffering from malaria [24, 55, 56]. However, Chaudhuri et al. [38] found significantly increased SOD and catalase activity in dogs infected with *B. gibsoni*. Additionally, *B. gibsoni*-infected erythrocytes in vitro showed increased values of SOD and glutathione reductase [30]. Differences in the pathophysiological effects of the parasite species or in the assay used for biomarker measurements could be the cause for these divergences. Generally, it is considered that antioxidant biomarkers are reduced in conditions associated with oxidative stress [57, 58]. Therefore, the significant reduction in antioxidant biomarkers found in diseased dogs in this study could be attributed to the consumption of antioxidants that act as “scavengers” of free radicals during the oxidative processes in the natural infection with *B. canis canis* in dogs. On the other hand, the increase in MDA concentration in dogs with babesiosis found in this research is consistent with the results of Crnogaj et al. [59] in dogs affected by babesiosis and implies its association with an increase in oxidative compounds.

Anaemia as a common feature of canine babesiosis has been investigated in numerous studies, yet its pathogenesis still remains questionable. Our study detected more pronounced decrease in antioxidant biomarkers (SOD, GPx and catalase) in dogs with moderate anaemia compared to those with mild anaemia. These findings contribute to the presumption that oxidative changes in dogs infected by *B. canis canis* are likely to be closely related to the pathogenesis of anaemia. Since there was just one severely anaemic dog, it was impossible to fully complete the comparison of anaemia with the investigated biomarkers, but it could be postulated that changes in severely anaemic dogs would be more prominent than those in moderately and mildly anaemic dogs. These results agree with those obtained in sheep with babesiosis and cattle suffering from theileriosis [31, 34, 53].

In the early stage of inflammation, it may be possible that endogenous substances such as SOD and reduced glutathione protect tissues from oxidative damage by ROS [60]. The imbalance of the redox state reflects an oxidative stress that may constitute a common pathway for life-threatening conditions and be responsible, at least in part, for tissue damage during a systemic response to injury [23]. Consumption of these substances of the antioxidant system after the persistence of SIRS may reinforce the oxidative stress after the initial inflammatory insult [60]. The existence of correlations between biochemistry parameters and antioxidants noted in this study may suggest that the severity of babesiosis is related to the degree of oxidative stress.

Although TAS did not show significant changes, individual antioxidants (SOD, catalase, GPx) showed significantly lower activity in dogs with MODS. This would imply that although measured antioxidant capacity does not show changes, in canine babesiosis, there are changes in selected components of the total antioxidants that can indicate the presence of MODS. This would be supported by the lack of correlation between TAS and the individual enzymatic antioxidants (SOD, catalase and GPx) in this study. Overall our results could be explained by the fact that the enzymatic antioxidants measured do not contribute greatly to the serum total antioxidant status [61]. Results of the current study concerning SOD, GPx and catalase activity as well as MDA concentration showed that dogs that developed MODS had more severe oxidative stress than dogs from other groups, which allows us to suppose that antioxidants investigated in this study have an influence on babesiosis severity as well as its outcome.

The authors of this research detected a strong negative correlation of the antioxidants measured (SOD, catalase and GPx) whereas a positive correlation of MDA with the lethal outcome of the disease. These results are in agreement with studies in critically ill people admitted to the hospital to intensive care units [35, 36] and they indicate a potential role of these biomarkers as prognostic indicators of disease severity and outcome in dogs suffering from babesiosis. However these data should be interpreted with caution since the dogs received different treatments.

## Conclusion

The results of this study demonstrated changes in antioxidant biomarkers associated with the presence of oxidative stress in dogs naturally infected with *B. canis canis*. These biomarkers could be used as indicators of disease severity and outcome in dogs suffering from babesiosis.

## Additional file

Additional file 1: Table S1. Descriptive statistics of biochemistry parameters in control and babesiosis group. Table S2 Descriptive statistics of haematology parameters in control and babesiosis group. (DOCX 14 kb)
