# Supplementary material for: Relation of antioxidant status at admission and disease severity and outcome in dogs naturally infected with Babesia canis canis
Source: BMC Vet Res. 2017 Apr 24;13:114. doi: 10.1186/s12917-017-1020-9 (PMC5402640; doi:10.1186/s12917-017-1020-9)
Supplement: Additional file 1: Table S1. — Descriptive statistics of biochemistry parameters in control and babesiosis group. Table S2 Descriptive statistics of haematology parameters in control and babesiosis group. (DOCX 14 kb) [file 12917_2017_1020_MOESM1_ESM.docx]

**Table S1 Descriptive statistics of biochemistry parameters in control and babesiosis group**

|  | **Control (N=30)** | **Uncomplicated (N=29)** | **Complicated (N=11)** | |
| --- | --- | --- | --- | --- |
|  |  |  | **One complication (N=6)** | **MODS (N=5)** |
| **Urea (mmol/L** | **5.6**  (4.4-6.3) | **7.0**  **(**5.2-8.6) | **10.1**  (6.0-22.9) | **22.0**  (18.9-30) |
| **Creatinine (µmol/L)** | **94**  (80- 101) | **87**  (73-102) | **92**  (66-195) | **190**  (106-325) |
| **Bilirubine µmol/L)** | **3.7**  (3.3-4.6) | **9.1**  (7.8-13.1) | **15.1**  (7.0-20.3) | **105.0**  (73.8-230.1) |
| **ALT (U/L)** | **42**  (34-52) | **57**  (40-97) | **229**  (42-641) | **101**  (65-840) |
| **AP (U/L)** | **44**  (29-69) | **121**  (96-151) | **217**  (118-482) | **338**  (101-798) |
| **CPK (U/L)** | **131**  (80-163) | **139**  (102-215) | **714**  (268-952) | **1300**  (717-2953) |

Median (25-75 percentile)

**Table S2 Descriptive statistics of haematology parameters in control and babesiosis group**

|  | **Control**  **(N 30)** | **Babesiosis (N 40)** |
| --- | --- | --- |
| **RBC (10^12^/L)** | **7.2**  (6.3-7.5) | **5.2**  (4.6-5.9) |
| **HCT (%)** | **50**  (46-53) | **37**  (32-43) |
| **WBC (10^9^/L)** | **10.9**  (9.2-12.1) | **5.3**  (4.0-7.2) |
| **PLT (10^9^/L)** | **280**  (232-345) | **28**  (19-36) |

Median (25-75 percentile)
